# TCDD-Induced Allosteric Perturbation of the AhR:ARNT Binding to DNA

**DOI:** 10.3390/ijms24119339

**Published:** 2023-05-26

**Authors:** Stefano Motta, Laura Bonati

**Affiliations:** Department of Earth and Environmental Sciences, University of Milano-Bicocca, 20126 Milan, Italy

**Keywords:** molecular dynamic, self-organizing maps, machine learning, toxicity, dioxins, AhR, 2,3,7,8-tetrachlorodibenzo-*p*-dioxin, ARNT

## Abstract

The aryl hydrocarbon receptor (AhR) is a ligand-activated transcription factor that mediates the biological and toxicological effects of structurally diverse chemicals, including halogenated aromatic hydrocarbons. In this work, we investigate the effects of the binding of the AhR prototypical ligand, TCDD, on the stability of the AhR:ARNT complex, as well as the mechanisms by which ligand-induced perturbations propagate to the DNA recognition site responsible for gene transcription. To this aim, a reliable structural model of the overall quaternary structure of the AhR:ARNT:DRE complex is proposed, based on homology modelling. The model shows very good agreement with a previous one and is supported by experimental evidence. Moreover, molecular dynamics simulations are performed to compare the dynamic behaviour of the AhR:ARNT heterodimer in the presence or absence of the TCDD. Analysis of the simulations, performed by an unsupervised machine learning method, shows that TCDD binding to the AhR PASB domain influences the stability of several inter-domain interactions, in particular at the PASA-PASB interface. The inter-domain communication network suggests a mechanism by which TCDD binding allosterically stabilizes the interactions at the DNA recognition site. These findings may have implications for the comprehension of the different toxic outcomes of AhR ligands and drug design.

## 1. Introduction

The aryl hydrocarbon receptor (AhR) is a ligand-activated transcription factor belonging to the basic helix-loop-helix Per-ARNT-Sim (bHLH-PAS) family of proteins [1,2]. It has attracted great attention since the 1970s as it acts as a chemosensory protein associated with the metabolism of various xenobiotics, the activation of which can lead to toxic outcomes [3,4,5]. However, in recent decades, significant experimental evidence has revealed that the AhR is also involved in the regulation of genes governing many pathophysiological processes, including cell growth and differentiation, cell cycle and migration, apoptosis, haematopoiesis, and carcinogenesis [6,7,8,9]. Particular attention has been paid to its key regulatory roles in the immune system [10,11,12]. Therefore, the AhR is now considered a key sensor and a promising drug target. The numerous cognate ligands of the AhR exhibit distinctive chemical structures. The most extensively studied exogenous ligands are the halogenated aromatic hydrocarbons (HAHs), which encompass the polychlorinated- dibenzo-*p*-dioxins, dibenzofurans, and biphenyls, and the polycyclic aromatic hydrocarbons (PAHs) [3,4,13]. One archetypal exogenous ligand of the AhR is the 2,3,7,8-tetrachlorodibenzo-*p*-dioxin (TCDD), a HAH with a high affinity for the AhR. TCDD and similar HAHs elicit diverse species- and tissue-specific biological and toxic effects. In addition to these well-known ligands, numerous natural, endogenous, and synthetic AhR agonists and antagonists have been discovered [14,15,16] and, recently, several ligands have been proposed for therapeutic applications and as selective AhR modulators (SAhRMs) [17,18,19,20]. Extensive research on the canonical AhR signalling pathway [1,2,21,22] has demonstrated that ligands bind to the receptor when it is part of a cytosolic multiprotein complex comprising heat shock protein 90 (hsp90), XAP2, and p23. Upon ligand binding, the AhR undergoes a conformational change, facilitating its translocation into the nucleus. Subsequently, the AhR displaces associated proteins through dimerization with the homologous ARNT (AhR nuclear translocator) protein, resulting in the formation of the AhR high-affinity DNA binding form. The ligand:AhR:ARNT complex binds to a specific DNA recognition site known as the dioxin-responsive element (DRE), ultimately leading to the transcription of target genes.

Structural information on the AhR N-terminal functional domains [23], including the bHLH and the PAS domains (PAS-A and PAS-B repeats), is particularly relevant to obtain a molecular description of the key events that regulate the AhR mechanism. The PAS-B repeat is involved in both ligand binding to the cytosolic AhR and, along with the bHLH, in binding to the chaperone hsp90 protein. The bHLH and the PAS domains are both involved in the AhR dimerization with the ARNT protein, and the bHLH is also responsible for binding to the DRE [1,2]. The lack of experimentally determined structures of these domains has hampered detailed mechanistic studies on the AhR for a long time. However, homology modelling has provided important contributions to the elucidation of the main events in the signalling pathway [24,25]. Models of the AhR PAS-B domain, based on the experimental structures of homologous bHLH-PAS proteins, have provided information on the ligand-binding cavities of AhRs of different species. The Hypoxia Inducible Factor 2α (HIF-2α) was the most used template, thanks to the highest degree of sequence identity with the AhR among all the reported PAS structures as well as to mechanistic similarities with the AhR. Molecular docking simulations of ligand binding to the modelled cavities have provided insights into differences in the binding of diverse agonists and antagonists, which were verified by site-directed mutagenesis and functional analysis of the mutants. In a similar way, the recently available X-ray structures of protein-protein and protein-DNA complexes of several bHLH-PAS systems [26,27,28] have opened the way to also modelling the AhR:ARNT dimer structure. In particular, our group previously developed two dimer models based on the CLOCK:BMAL [26] and on the HIF-2α:ARNT [27] X-ray structures. The latter model showed consistency with several mutagenesis experiments at the inter-domain interfaces [29]. Finally, a few years ago, the structure of the AhR:ARNT dimer, limited to the region including the core of the PAS-A domains, the bHLH domains, and the DNA-recognition site, was determined by X-ray crystallography [30,31]. This finding can bring great advancements to the molecular understanding of the AhR mechanism. However, only with a reliable structural model of the overall quaternary structure of the dimer, including the PAS-B domains and the long mobile loops of the PAS-A domains, not yet resolved, will it be possible to completely define the inter-domain interactions. A complete model would also open the possibility of analysing the effects of ligands on the structure and dynamics of the complex, thus providing information on both the ligand-induced transformation of the AhR and the DNA binding. In a previous study, we were able to unveil how ligand-induced perturbations can dynamically propagate through the HIF-2α:ARNT structure and affect dimerization by comparing the dynamics of the dimer in the presence and absence of an inhibitor [32].

On these grounds, in this study, a complete structural model of the AhR:ARNT dimer is proposed. It includes the bHLH and PAS-A domains derived from the experimental structure [30], the PAS-B domains of the two partners that are obtained by homology modelling (based on the HIF-2α:ARNT template structure), and the unresolved conformations of the PAS-A loops that are modelled de novo and refined through Molecular Dynamics (MD). The dimer structure in complex with the TCDD is also determined through molecular docking. To highlight the effects of the ligand on the dynamics of the complex, 3 × 1 μs-long MD simulations are performed for both the apo (ligand-free) and holo (TCDD-bound) forms of the complex. The comparison between these simulations allowed us to identify the TCDD effects on the stability of the quaternary arrangement, to reveal the TCDD-induced perturbations on the correlated motions of the domains, and to determine allosteric communication, thus elucidating how ligand binding influences the interactions between the dimer and the DNA.

## 2. Results

### 2.1. Structural Model of the AhR:ARNT:DRE Complex

The X-ray structure of the AhR:ARNT:DRE complex (PDBID: 5NJ8) includes the bHLH-PASA region of both proteins, but lacks their PASB domains and the long PASA loops. Since no template structures are available for the latter, we used two different strategies to model the core and the loop regions.

**Figure 1:**
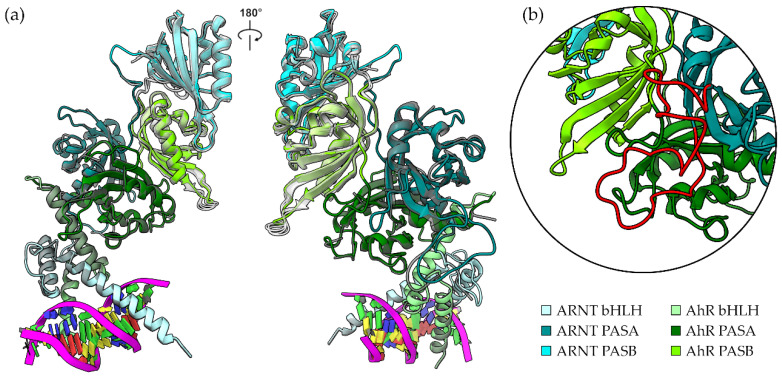
Model of the AhR:ARNT:DRE complex. (**a**) Quaternary architecture of the core model. Proteins are represented as cartoons and domains are marked with different colours. The structure 5nj8, for the bHLH-PASA, and the template structures 4zp4, 4zqd, and 6e3u for PASB, are shown as semi-transparent grey cartoons for comparison. (**b**) Close-up on the region of the ARNT FG-loop that interacts with AhR (red cartoon).

At first, starting from the X-ray structure (5NJ8), we completed the dimer by adding the two PASB domains and the connecting linkers by homology modelling, with the HIF-2α:ARNT complexes as templates. The model was generated using the MODELLER software package, by adopting a multi-template strategy (see Section 4), i.e., by considering the apo and two inhibitor-bound forms of HIF-2α [27,33] to better describe the plasticity of the binding cavity in the PASB. The secondary structure elements of a typical PAS fold are presented in Figure S1a. The core model of the entire AhR:ARNT dimer shows good stereo-chemical quality, as assessed by PROCHECK, with 85.8% of residues located in the most favoured regions of the Ramachandran plot and 0.9% in the disallowed regions. The overall arrangement of the six domains closely resembles the one observed in the HIF-2α:ARNT dimer (Figure 1a), with an RMSD of 0.94 Å from the 4ZP4 structure (calculated on 421 out of the 529 total aligned residues, see Section 4).

Consequently, this new model also matches with the homology model we previously developed for the bHLH-PASA-PASB region of the AhR:ARNT dimer on the basis of the HIF-2α:ARNT template structure (4ZP4) [29], with an RMSD of 1.73 Å (on 535 out of 604 total aligned residues).

In the second stage, the long and flexible loops of both the AhR and the ARNT PASA domains were modelled using the MODELLER loop modelling routine, and they were further refined using an external simulated annealing approach (see Section 4). In this latter simulation, the long loops evolved to stable conformations, suitable for the subsequent MD simulations. The nomenclature of the PAS loops and the interdomain linker employed in this study are presented in Figure S1b. In the final structural model, we observed that the long ARNT FG loop in the PASA domain contributes to stabilizing the dimeric structure. It mainly interacts with the AhR PASA-PASB linker (Figure 1b), with a computed change in the Solvent Accessible Surface Area (ΔSASA) of the ARNT FG loop of 502 Å^2^. The same ARNT loop has been shown to stabilize the dimeric form of the HIF2α:ARNT complex [32]. Overall, the interdomain interfaces described in previous works are preserved (Figure S2)

In this study, the binding mode of TCDD was predicted using the Glide XP docking method. Two different binding poses (Figure S3) were obtained, which differed for a modest (about 3.5 Å) translation within the binding cavity. We observed the same variance in the TCDD binding mode in a previous study [25], where the docking pose of TCDD in the modelled PASB was also subjected to refinement by short MD simulations. The pose with the lowest Emodel score was selected for further simulations of the AhR:ARNT dimer in the holo form.

### 2.2. TCDD Binding Influence on the Stability of the Quaternary Arrangement

**Figure 2:**
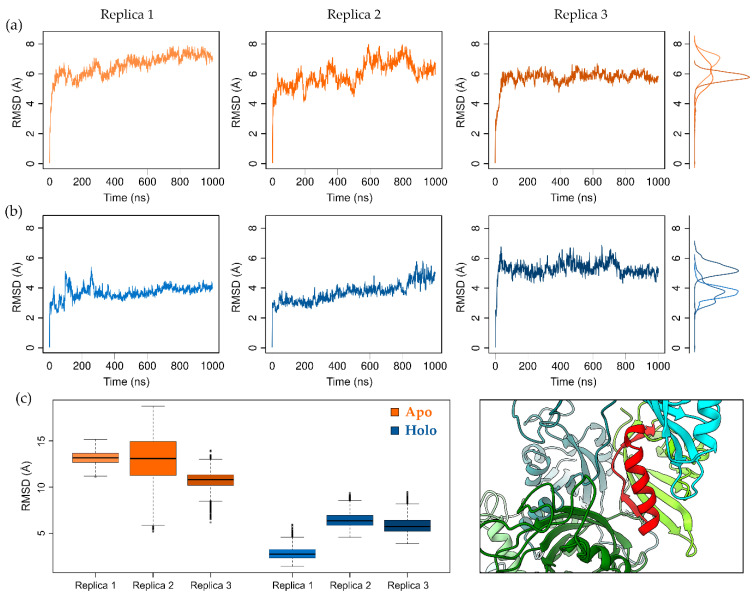
Geometric deviation from the starting equilibrated structure during MD simulations. Plots of the RMSD for (**a**) the apo systems and (**b**) the holo (TCDD-bound) systems. RMSD were computed on Cα atoms of proteins, excluding the regions of the flexible PASA loops. The densities of RMSD values are reported at the right margin. (**c**) Displacement of AhR PASB Fα and Gβ shown as boxplots of RMSD values computed on Cα atoms of this region after alignment on Cα atoms of AhR PASA domain. AhR PASB Fα and Gβ are shown as a red cartoon in the 3D representation, while AhR PASA is shown as a forest-green cartoon.

The system in its ligand unbound (apo) and bound (holo) forms was simulated through three MD replicas of 1 μs each. The RMSD plots of the core domains show a rapid increase in the RMSD during apo simulations, while in the holo simulations, the protein remains closer to the starting geometry (Figure 2a,b). During apo simulations, the system deviates by approximately 5–8 Å from the starting conformation, whereas smaller deviations are observed in the TCDD-bound simulations (about 2–4 Å in replicas 1 and 2, and 4–6 Å in the third replica).

To investigate the origin of this different behaviour, we computed the principal component of the motion. A comparison of the apo and holo simulations revealed a discrepancy along the first eigenvector (Figure S4a), which represents the opposite rotation of the PASB with respect to the PASA domain in both the proteins (Figure S4b). The region mainly involved in this perturbation turned out to be the AhR PASA-PASB interface, which deviates from its starting conformation. To quantify the magnitude of the displacement between the two domains, we aligned the frames of the simulations on the AhR PASA Cα atoms and computed the RMSD of the Cα atoms belonging to two elements of the PASB at the interdomain interface (the AhR PASB F helix (Fα) and G strand (Gβ), Figure 2c). We found that apo simulations largely deviate from the starting geometry due to a shift in the PASB orientation.

Therefore, our simulations indicate that the TCDD ligand influences the stability of the quaternary architecture of the AhR:ARNT dimer, by strengthening the interactions at the AhR PASA:PASB interface.

### 2.3. Analysis of Interdomain Correlated Motions Reveals Apo/Holo Differences

**Figure 3:**
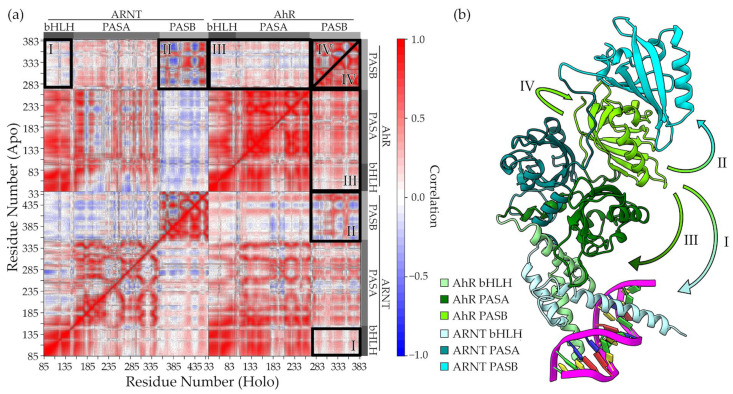
Distance cross-correlation matrices for apo and holo simulations. (**a**) Correlation matrices (upper triangular for apo, lower for holo) with domains labelled on the sides. Four regions are highlighted with black rectangles and labelled with romans numbers. (**b**) Intra- and inter-domain correlations highlighted in panel (**a**) are represented with arrows. Protein domains are coloured with different colours.

The analysis of per-residue correlated motions, performed by means of the distance cross correlation matrix [34,35] (DCCM), revealed both short- and long-distance effects triggered by the ligand (Figure 3). Particularly, the motions of the AhR ligand-binding domain (PASB) were significantly influenced by the presence of the ligand. At first, we detected a perturbation in the AhR PASB intradomain correlation (box IV in Figure 3a, and in an enlarged representation in Figure S5). In the apo simulations, some low-correlated or anti-correlated regions could be spotted on the map. These included the correlation between AhR PASB Fα-Gβ with the helical bundle (particularly Cα and Dα) and the GH loop of the same domain. Conversely, in the holo simulations, the domain appeared more compact, with high residue-correlation values also observed for these regions of the map.

The compactness of the domain was further assessed using the community detection method [36], which provides a clear indication of groups of residues moving in a concerted manner (Figure S6). In apo simulations, residues of the AhR PASB domain were assigned to different communities, while a unique community was found in holo simulations. This analysis confirmed the stabilizing effect of TCDD on the AhR PASB domain.

Moreover, we detected different correlations of motions of the AhR PASB with those of other domains between the apo and holo simulations. Correlation with the ARNT bHLH (box I in Figure 3a) and with the other domains of AhR (box III in Figure 3a) was higher in holo than in apo simulations, indicating that TCDD may increase the AhR PASB communication with these N-terminal domains. This effect may represent the way the AhR PASB can communicate with the DNA-binding region. The correlation of the AhR PASB with the ARNT PASB motions, on the other side, also presents relevant differences between the apo and holo simulations. In fact, higher values in both the correlated and anticorrelated motions can be observed in apo simulations (box II in Figure 3a). Together these data suggest that the AhR PASB is highly connected with the C-terminal end of the dimer (ARNT PASB domain) in apo simulations, while TCDD increases the PASB internal stability and its connection with the N-terminal side of the dimer (DNA-binding region), thus reducing its connection with the C-terminal end.

### 2.4. Binding of TCDD Allosterically Influences Interactions with DNA

The classical ligand-activated mechanism of the AhR culminates in the binding of the ligand:AhR:ARNT complex to its specific DNA recognition site, leading to transcription of the target genes and the production of a wide variety of biological and toxicological effects depending on the ligand [22]. Therefore, our main aim was to investigate whether TCDD binding can cause alterations in the interactions of the complex with DNA.

To evaluate the subtle differences between the apo and holo simulations at the complex-DNA interfaces, we monitored the distances between certain charged residues of the protein complex at the interface and their interacting partners in the DNA, as well as the distances within two ARNT intramolecular salt bridges (details in the Section 4), for a total of 25 distances (Table S1). To this end, we used Self-Organizing Maps (SOMs) (see Section 4), a type of artificial neural network that produces an explicit visual representation of data, including protein features extracted from MD simulations, on a two-dimensional map to highlight the similarities or differences among them. Here, we applied SOMs to detect differences in the above interatomic distances sampled during our apo and holo simulations.

**Figure 4:**
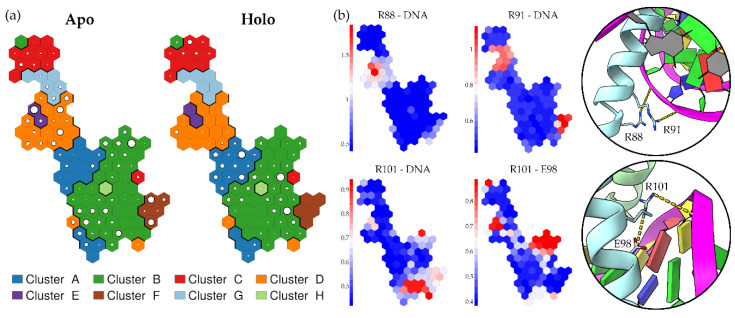
SOM trained with selected salt bridge distances at the protein-DNA interface. (**a**) SOM clustered at the neuron level. The population of each neuron is shown for both the apo and holo simulations by the size of white circles within the neuron. (**b**) SOM neurons coloured according to the values of four selected inter-residue distances that displayed different behaviours in the apo and holo simulations. A 3D representation of these distances is shown within the circles to the right.

A 10 × 10 toroidal SOM was trained with the set of 25 distances, derived from both the apo and holo simulations. The resulting SOM displayed neurons that are exclusively or mostly visited by apo simulations and others by holo simulations (Figure 4a). Apo simulations sampled clusters D and G, which were less sampled by holo simulations. Holo simulations, in contrast, populated clusters A and F. Furthermore, within cluster B, the biggest cluster of the SOM, it was possible to detect some differences in the population of neurons from the two sets of simulations.

To detect features that mostly characterize regions of the map that are exclusive for one group of simulations, we coloured the SOM neurons according to the average value of some monitored distances. Distances that behave differently in apo and holo simulations should assume different values in the regions of the map that are exclusive for one type of simulation. We detected a set of four distances that assumed different values in these exclusive regions, thus discriminating apo from holo simulations. All of them were distances at the ARNT bHLH—DNA interface and suggested the presence of altered interactions of ARNT with DNA in this region when the dimer is bound to the TCDD (Figure 4b). All the above distances were lower in holo simulations, indicating the formation of strong interactions in the presence of TCDD, while they were higher in the apo simulations. The four distances are related to the salt bridges between residues R88, R91, and R101 and DNA phosphate groups, as well as to the intramolecular salt bridge between R101 and E98 (not present at the beginning of the simulations but formed during some of them).

These differences suggest a TCDD-induced alteration of the interactions at the ARNT:DNA interface. In fact, apo simulations tend to sample regions characterized by higher distances, indicating that some interactions are lost or not formed without TCDD.

**Figure 5:**
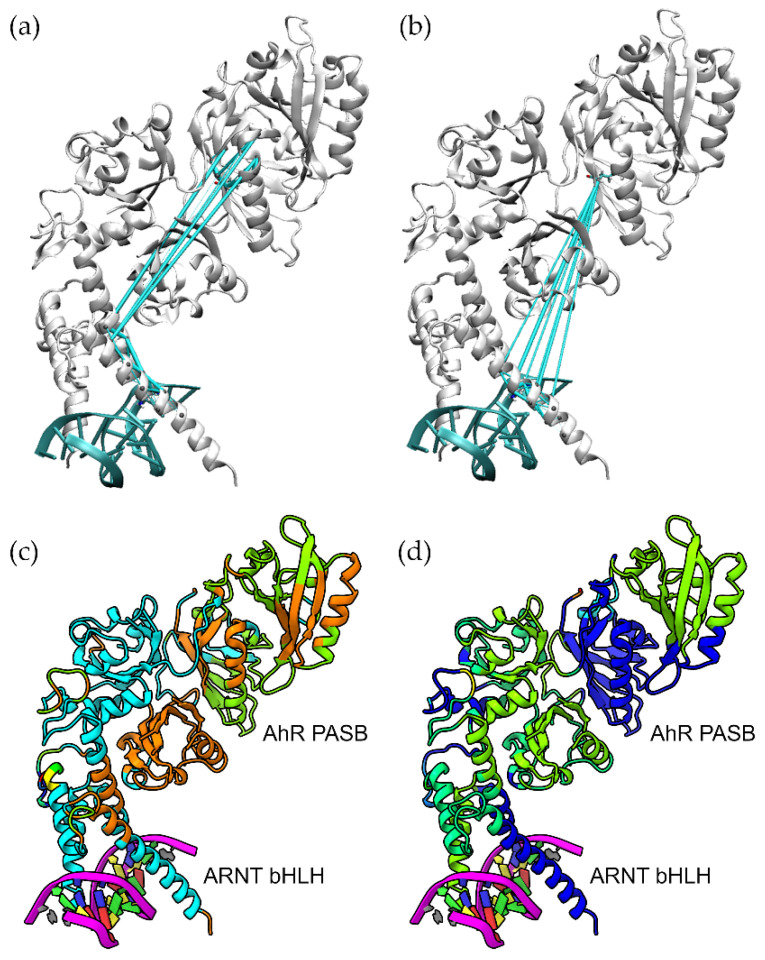
Link between AhR PASB and ARNT bHLH at the DNA interface. A 3D representation of the optimal and suboptimal paths connecting the two regions in apo (**a**) and holo (**b**) simulations. Community detection for the apo (**c**) and holo (**d**) simulations. In holo simulations both AhR PASB and ARNT bHLH fall within the blue community.

To further investigate the connection between the presence of the ligand and the perturbation at the level of the ARNT:DNA interface, we built a graph where nodes were residues and edges were the correlations of the DCCM matrix. From this graph, we computed the optimal and sub-optimal paths [37] between a region of the AhR PASB (Fα) and the ARNT bHLH (Figure 5a,b). In the case of the holo network, a strong connection between AhR-PASB and ARNT-bHLH exists, suggesting that the local perturbation induced by the ligand within the PASB domain could propagate to the bHLH domain. This was further confirmed by the community analysis on the whole protein (Figure 5c,d) that showed the AhR ligand-binding domain within the same community of the ARNT bHLH domain in holo simulations, differently from the apo simulations.

## 3. Discussion

In this work, we investigated the effects of the binding of the AhR prototypical ligand, TCDD, on the stability of the AhR:ARNT:DRE complex, as well as the mechanism through which ligand-induced perturbations propagate to the DNA recognition site responsible for gene transcription. We proposed a computational approach involving the comparison between MD simulations of the apo and of the ligand-bound complexes. Previously, we employed a similar strategy to propose a model for the inhibition of the HIF-2α:ARNT dimerization by the 0X3 ligand [32]. In that study, comparisons of the dynamics of the apo and inhibitor-bound complexes revealed that the perturbation induced by the ligand in the HIF-2α PAS-B dimerization interface allosterically propagates to the PAS-A:PAS-A interface, thus destabilizing one of the most important regions for dimer association.

To perform the proposed study, a reliable structural model of the overall quaternary structure of the AhR:ARNT:DRE complex was required. The unique X-ray structure available, which includes the bHLH-PASA region of the dimer bound to the DRE [30], served as the appropriate base. Then, the complete model was developed by adding the PAS-B dimer region and the interdomain linkers through homology modelling (using multiple HIF-2α:ARNT template structures) as well as the unresolved PAS-A loops by de novo modelling and MD refinement. The proposed model shows a very good agreement with a previous one [29] we entirely developed through homology modelling on the basis of the HIF-2α:ARNT dimer structure [27] (the template structure with the highest identity available at that time). The dimerization mode, common to both models, has been supported by several experimental studies [29]. Some of these data validate the arrangement of the two partners in the crystallographic bHLH-PASA region; furthermore, residues that lie at the modelled protein-protein interfaces were shown to be critical for dimerization based on mutagenesis and functional analysis studies. The D217 and C265 ARNT PAS-A residues [38] that were shown to affect AhR:ARNT dimerization were at the interface with the PASA-PASB linker of the AhR. Mutations of Y316 and I324 in the helical region of the AhR PAS-B and of a few ARNT residues in the PAS-B β-sheet (F446, N448, E455, I458) were shown to disrupt AhR:ARNT dimerization, and these residues belonged to the modelled AhR:ARNT PASB-PASB interface [39].

In the last few months, the first experimental structures of the AhR PAS-B have been published, and they support our modelling efforts for this domain. The structure of the *Drosophila* AhR (dAhR) PAS-B has been determined by X-ray crystallography both in the apo and *α*-naphtoflavone-bound forms [40]. The authors reported that, compared to the HIF-2α PASB, the apo form shows a similar overall fold, with major differences in a C-terminal element. Accordingly, the mAhR PAS-B model developed in this work, as well as our previous models of the isolated AhR PAS-B [24,25,41] similarly based on the HIF-2α template (all not including the C-terminal tail), shows a good agreement with the dAhR PASB (RMSD = 0.60 Å calculated on 85 out of the 103 total aligned residues). A small difference is observed in the Eα helix region (see Figure S1a), which is arranged as a loop in the X-ray structure. In the same paper, a structure of the dAhR:mARNT dimer limited to the PAS-B region was also proposed [40] and our model shows a good agreement with that architecture. Moreover, important structural information on the AhR was obtained by the very recent 2.85 Å cryo-electron microscopy (Cryo-EM) structure of the human cytosolic complex comprising the chaperone Hsp90 and the co-chaperone XAP2 proteins, along with an AhR portion including the PASB bound to the endogenous indirubin ligand, a fragment of the AhR PASB-PASA linker, and a carboxy-terminal extension of the PAS-B [42]. Furthermore, the Cryo-EM structure of the murine cytosolic Hsp90-XAP2-AhR-p23 complex including the AhR PAS-B domain has been determined [43]. The AhR PAS-B fold modelled in this work shows consistency with those included in both the Cryo-EM structures (RMDS = 1.20 Å and 1.54 Å for the aligned residues, from the former and the latter structure, respectively).

On this basis, MD simulations were performed to compare the dynamic behaviour of the AhR:ARNT heterodimer in the presence or absence of the AhR prototypical ligand TCDD. The simulations show that TCDD binding affects the stability of some interdomain interactions, in particular at the AhR PASB interface with both the PASA domains. The TCDD-bound dimer appears more stable than in the apo form, with the PASB domain fluctuations significantly reduced. Consequently, the motion of the AhR PAS-B in the holo complex appears more correlated with the motion of the N-terminal domains, as shown by the distance cross-correlation analysis. This effect is different from what was observed from the simulations of the homologous HIF-2α:ARNT heterodimer, in which the altered interfaces were the heteromeric PASA/PASA and PASB/PASB [32]. In that case, however, ligand binding was shown to inhibit the dimerization of HIF-2α with its partner ARNT, whereas, in the case of AhR, the TCDD role is the activation of the DNA transcription mechanism. Following these results, the ligand binding to the PASB domain of bHLH-PAS proteins appears to have a regulatory role in the PASB flexibility and in the inter-domain communication within the dimeric complex.

Moreover, given that it has been demonstrated that diverse ligands can differentially affect AhR activation and the transcription mechanism, it is conceivable that they produce different effects on DNA binding [22,44]. To understand these long-range effects, we used SOMs to evaluate subtle differences between the apo and holo simulations at the complex-DNA interface. This analysis revealed distinct networks of interactions between a group of ARNT arginine residues involved in the stabilization of DNA binding for the apo and holo systems. The motion of these residues was found to be strongly correlated with that of the AhR PASB domain in holo simulations, supporting the hypothesis of a long-range effect of the ligand.

In conclusion, the results presented here support a model of TCDD-dependent activation in which the mobility of the AhR PASB domain is reduced by the ligand, thereby affecting its interdomain interactions. This effect allosterically propagates to the interface between the AhR:ARNT complex and the DNA, providing a mechanism for the ligand modulation of gene transcription. Future studies will be directed at identifying differences in both the dynamic behaviour and the DNA interactions of the AhR:ARNT complex when bound to different AhR ligands that exert differential modulation of the DNA activation mechanism.

## 4. Materials and Methods

### 4.1. Homology Modelling

The structure of the murine AhR:ARNT dimer was obtained starting from the dimer X-ray structure including the murine ARNT (mARNT) and the human AhR (hAhR) and encompassing the bHLH-PASA region [30] (PDB code: 5NJ8). The structure was completed using a multi-step strategy (Figure S6). At first (step 1), the mAhR:mARNT core model (without the long PASA loops) was obtained by homology modelling. We employed the MODELLER 9v8 [45] software, using the hAhR:mARNT structure (5NJ8) to model the bHLH-PASA region and some structures of the mARNT:mHIF-2α as templates for the PASB region and the interdomain linkers. Given that multiple X-ray structures for the ARNT:HIF-2α complex exist, we used a multi-template strategy including the depositions of the dimer in the apo form [27] (4ZP4), bound to an HIF2α antagonist [27] (4ZQD) and to an agonist [33] (6E3U). Based on the residues available in the selected template structures, the modelled region included the amino-acids: mAhR: 33–384 and mARNT: 85–464. The optimal structure of each AhR:ARNT dimer model was selected among 1000 models generated by MODELLER according to the best value of the distance-dependent statistical potential (DOPE) score [46]. As a second step, the missing residues in the PAS-A loops of the AhR:ARNT X-ray structure 5NJ8 (mAhR residues: 175–202; 245–254 and mARNT residues: 229–258; 270–301; 317–333) and the AhR bHLH/PASA linker (mAhR residues: 88–106) were built using the MODELLER loop modelling routine, at the most accurate refine.slow_large level. Even at this level, given the length of the missing loops, the quality of the prediction is expected to be poor. Given the intrinsic dynamical behaviour of this region, we decided to further relax the conformation of the loops by performing a simulated annealing protocol on the model with the best DOPE score (step 3, see Section 4.4).

### 4.2. System Preparation

The DRE region of the DNA included in the 5NJ8 structure was added to the dimer model. Subsequently, the entire model underwent pre-processing for simulation using Schrödinger’s Protein Preparation Wizard tool [47]. The pre-processing steps included the addition of hydrogen atoms, the removal of all water molecules, the introduction of C and N terminal capping, the assignment of disulfide bonds, and the determination of residue protonation states using PROPKA. Each system was then solvated in a cubic box filled with TIP3P water molecules and neutralized by adding Na^+^ ions, employing the GROMACS preparation tools [47,48]. A minimal distance of 14 Å was set between the protein and the boundaries of the simulation box. The simulations were performed using GROMACS 2018.1 with the Amber14sb force field [49]. Parameters for the TCDD molecule were obtained from the GAFF force-field [50], which were calculated using the restricted electrostatic potential (RESP) method [51] at the ab initio HF/6-31G* level, after optimization of the ligand at the same level.

### 4.3. PASA Loop Simulated Annealing

The system was first subjected to 2000 steps of steepest descent energy minimization with high positional restraints (10,000 kJ mol^−1^ nM^−2^) on all protein and DNA atoms, except for unresolved PASA loops. These restraints were maintained during all of the subsequent steps to preserve the X-ray structure in the resolved regions [30]. The system was then heated from 0 to 300 K during a 1 ns NVT simulation, followed by 3 ns of NVT simulation at 300 K. The temperature was controlled using a Berendsen thermostat [52] with a coupling constant of 0.2 ps. A timestep of 1 fs was used during these preliminary steps. The system was then subjected to simulated annealing (maintaining restraints on resolved atoms). The temperature was increased up to 750 K in 5 ns, the temperature was then kept constant for 5 ns, and finally, the system was slowly cooled down to 300 K during 15 ns. Finally, 5 ns of MD simulation at 300 K was performed. The full protocol is schematized in Figure S7.

### 4.4. Molecular Docking

The TCDD structure was drawn in Maestro and then prepared with the LigPrep [53] utility in the Schrödinger suite. The ligand structure was optimized using MacroModel and the OPLS3 [54] force field, by employing implicit water as the solvent. To generate the holo AhR:ARNT model, molecular docking was conducted using Glide XP [55] (extra precision) included in the Schrödinger suite 2016–3. This docking method incorporates a hierarchical set of filters to explore potential binding site locations for the ligand and incorporates a flexible treatment of the ligand. For this study, default parameters were utilized to set up the grids, and the binding box, with a side length of 10 Å, was positioned at the centre of mass of the PASB domain.

### 4.5. Molecular Dynamics Simulations

The complete apo and holo models, including both the regions obtained by the homology models and the ones optimized by simulated annealing, were subjected to MD simulations. At first, 2000 steps of steepest descent energy minimization with high positional restraints (2000 kJ mol^−1^ nM^−2^) on backbone atoms were performed. Following that, a 2 ns NVT MD simulation was performed to gradually heat the system from 0 to 100 K, with restraints gradually reduced to 500 kJ mol^−1^ nM^−2^. Subsequently, the system was further heated to 300 K over a 4 ns NPT simulation, with restraints lowered even further (250 kJ mol^−1^ nM^−2^). The system then underwent a 10 ns equilibration period in an NPT simulation, with backbone restraints gradually reduced to 150 kJ mol^−1^ nM^−2^. This was followed by a second 10 ns equilibration stage, with restraints lowered to 50 kJ mol^−1^ nM^−2^. During the production run at 300 K, all of the restraints were removed. Three independent production replicas were performed for both the apo and holo forms, each running for 1 μs. During the NVT simulations, the temperature was controlled using the Berendsen thermostat [52] with a coupling constant of 0.2 ps. In the NPT simulations, a V-rescale thermostat [56] with a coupling constant of 0.1 ps was used to control the temperature, while the pressure was maintained at 1 bar using a Parrinello-Rahman barostat [57] (coupling constant of 2 ps). A time step of 2.0 fs was used, together with the LINCS [58] algorithm, to constrain bonds involving hydrogen atoms. Long-range electrostatic interactions were treated using the particle mesh Ewald method, [59] with cut-off distances set at 12 Å. We want to underline the choice of a more extended (16 ns) multistage equilibration protocol compared to the protocol previously used for similar cases [32,60], aimed at improving the starting point of the simulation, given that some portions of the system have been derived from homology modelling.

### 4.6. Self-Organizing Maps

SOMs are a type of unsupervised artificial neural network where neurons are arranged in a grid map, enforcing topological relationships [61]. Multidimensional input data can be effectively visualized in a low-dimensional representation using a SOM. Each neuron is a feature vector with the same dimension as the input data vectors. The training of the map is an iterative process in which neurons adapt to the input data, creating a map that represents the input data. SOMs have been used for the analysis of biomolecular simulations of various types [62,63,64,65,66,67]. In this work, we used a 10 × 10 toroidal SOM (with periodicity across the boundaries) with a hexagonal lattice shape. The input features to train the SOM are a set of distances at the protein-DNA interface (Table S1). Distances were computed every 100 ps from both the apo and the holo MD simulations. In the second step, the neurons are further grouped in a small, but representative, number of clusters by agglomerative hierarchical clustering using Euclidean distance and complete linkage. The optimal number of clusters, eight, was selected based on the Silhouette profiles (Figure S8). Since the SOM used in this work was toroidal, visual interpretation could have been difficult. To overcome this issue, we applied a workflow to optimize visualization, as also proposed in other works [64]. In this case, we started the assignment of neuron positions from the most populated neuron and assigned neurons to maintain the border neurons that were more dissimilar to each other. All of the analyses were performed in the R statistical environment [68] using the kohonen package [69,70].

### 4.7. Analysis of Correlated Motions and Long-Distance Communication

To identify protein segments exhibiting correlated atomic motions, a correlation network analysis [34,35] was conducted using the Bio3D library in R [36,71]. The displacement between the Cα atom pairs was used to estimate the pairwise residue cross-correlation coefficients [72] and these were collected in the DCCM matrix. Utilizing the cross-correlation matrix, a weighted graph was generated, assigning each residue as a node and setting edge weights as the correlation values among residues. Positive weights indicated correlated motions, while negative weights indicated anti-correlated motions. Shortest and suboptimal path analysis [36], conducted on the 50 shortest detectable paths, was used to highlight differences in interdomain communication in the apo and holo simulations. Community detection was carried out using the random walker algorithm [36], only considering edges with absolute correlation values greater than 0.5. A similar approach had already been proposed for the study of a similar system [32].

### 4.8. RMSD Calculation and Visualization

RMSD values on static structures were computed using the align function implemented in Pymol [73]. This approach performs a sequence alignment followed by a structural superposition, and then carries out a few cycles of refinement in order to reject structural outliers found during the fit. In this way, it compares proteins with different numbers of residues. The RMSD on MD trajectories was computed using gromacs [48] rms function. Visual representation was obtained using ChimeraX [74] software, except for panels a and b of Figure 5, which were generated with VMD [75]. All plots and statistical analyses were performed using the R statistical environment [68].

## Data Availability

The model structure and the MD simulation files are available at: Motta, Stefano; Bonati, Laura (2023), “Data for: TCDD-induced allosteric perturbation of the AhR:ARNT bind-ing to DNA”, Bicocca Open Archive Research Data, V1, https://doi.org/10.17632/y48yp2tx9p.1. Other data presented in this study are available on request from the corresponding author.

## Supplementary Materials

The following supporting information can be downloaded at: https://www.mdpi.com/article/10.3390/ijms24119339/s1.
