# Supplementary material for: TCDD-Induced Allosteric Perturbation of the AhR:ARNT Binding to DNA
Source: Int J Mol Sci. 2023 May 26;24(11):9339. doi: 10.3390/ijms24119339 (PMC10253319; doi:10.3390/ijms24119339)
Supplement: Supplementary file 1 [file ijms-24-09339-s001.zip › ijms-2357785-supplementary.pdf]

Article

TCDD-Induced Allosteric Perturbation of the AhR:ARNT Binding to DNA

Stefano Motta[1] and Laura Bonati[1,*]

Department of Earth and Environmental Sciences, University of Milano-Bicocca, Piazza della Scienza 1, 20126 Milan, Italy

* Correspondence: laura.bonati@unimib.it; Tel.: (+39) 0264482821

Supplementary Materials

Figure S1. Nomenclature of the PAS domain secondary structure elements and connecting elements.
Figure S2. 3D structure of the monomeric units that are part of the complex and interdomain interfaces.
Figure S3. Docking poses for the TCDD molecule within the AhR PASB domain obtained with Glide XP.
Figure S4. Principal component analysis..
Figure S5. Representation of communities for residues belonging to the AhR PASB domain.
Figure S6. Multiple sequence alignment for the AhR:ARNT complex and the templates used for the homology modelling steps.
Figure S7. Plot of the simulated annealing protocol.
Figure S8. Plot of the average silhouette width against the number of clusters.
Table S1. Atoms involved in distances used to train the SOM.

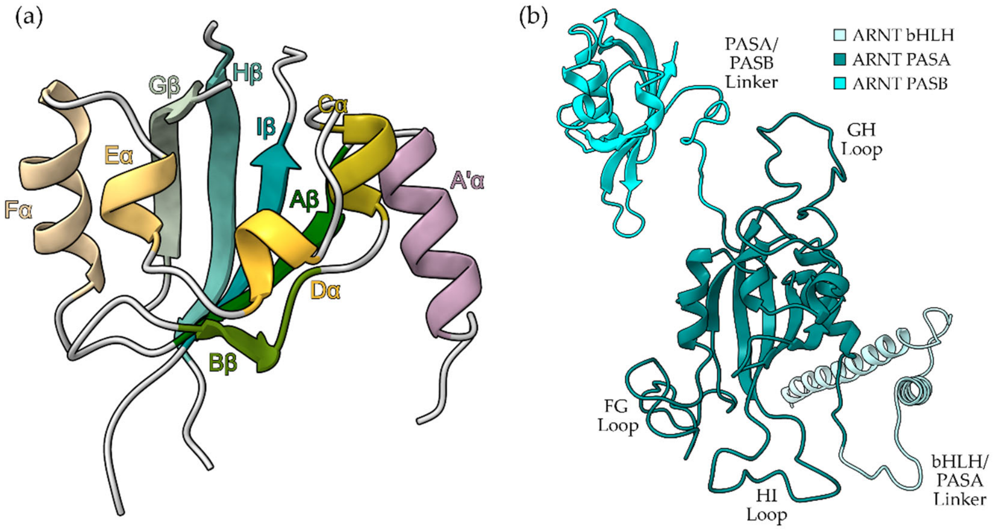

Figure S1. Nomenclature of the PAS domain secondary structure elements and connecting elements. (a) Typical PAS-domain fold represented using the three-dimensional structure of the ARNT PAS-A domain. Secondary structure elements are a five-stranded antiparallel β-sheet (the N-terminal Aβ, Bβ and the C-terminal Gβ, Hβ, and Iβ) flanked by a long α-helix (Fα, called "helical-connector") and several shorter α-helices (Cα, Dα, and Eα, often called "helical bundle"). The PAS-A and PAS-B folds mainly differ in the length of connecting loops, and in the presence of an additional N-terminal α-helix (A'α) in the PAS-A. (b) bHLH-PAS region of the ARNT protein with the PAS-A loop modelled. Nomenclature for the PAS-A loops and the interdomain linker is reported.

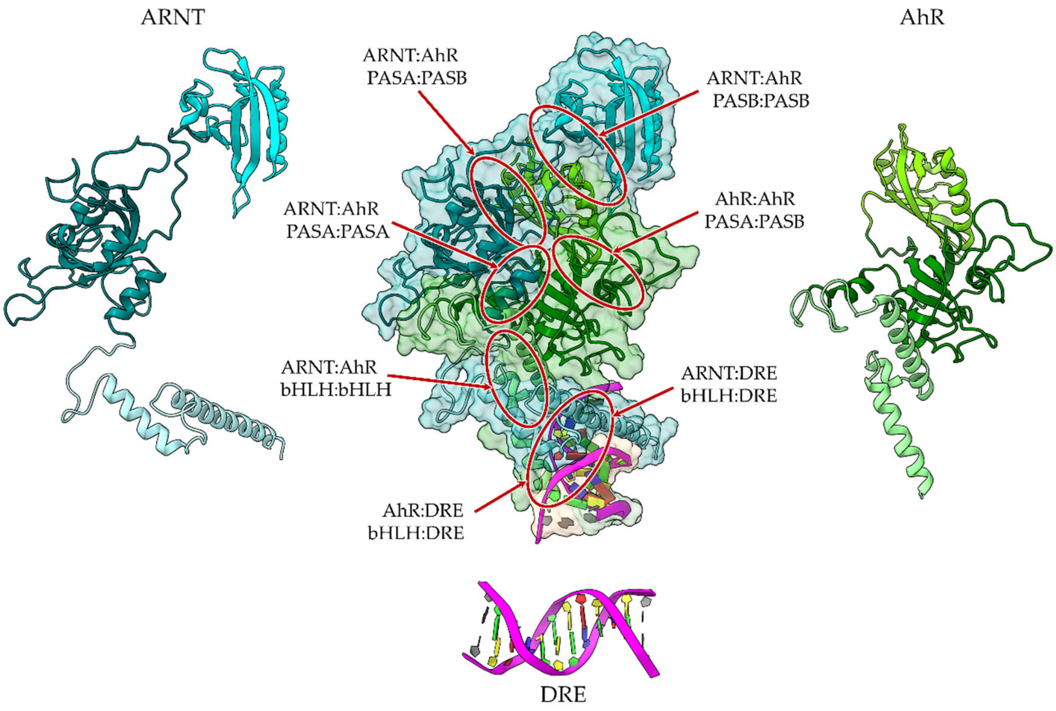

Figure S2. 3D structure of the monomeric units that are part of the complex and interdomain interfaces. ARNT protein is shown in cyan, AhR in green and DRE as magenta cartoons. Interdomain interfaces are evidenced with red circles.

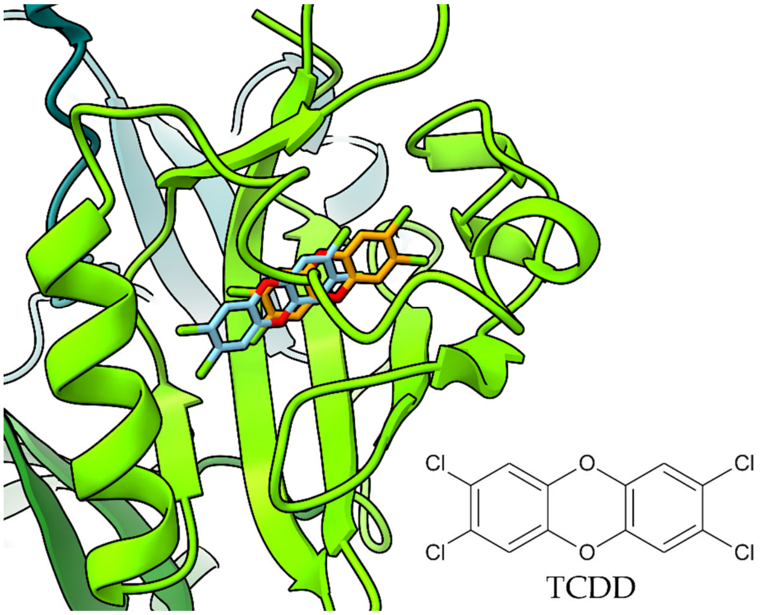

Figure S3. Docking poses for the TCDD molecule within the AhR PASB domain obtained with Glide XP. The best pose according to the Emodel score is represented in cyan, while the second pose in orange. The structure of the TCDD is reported in the bottom right corner.

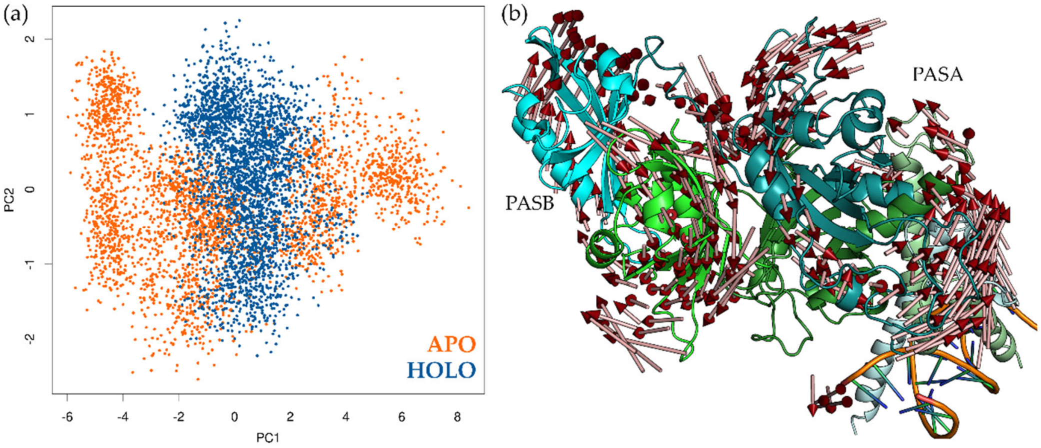

Figure S4. Principal component analysis. (a) Plot of principal component 1 (PC1) and principal component 2 (PC2) computed on protein Cα atoms. Frames from apo simulations are reported in orange and frames from holo simulations in blue. (b) Modvector representation of the first eigenvector. Image prepared in Pymol.

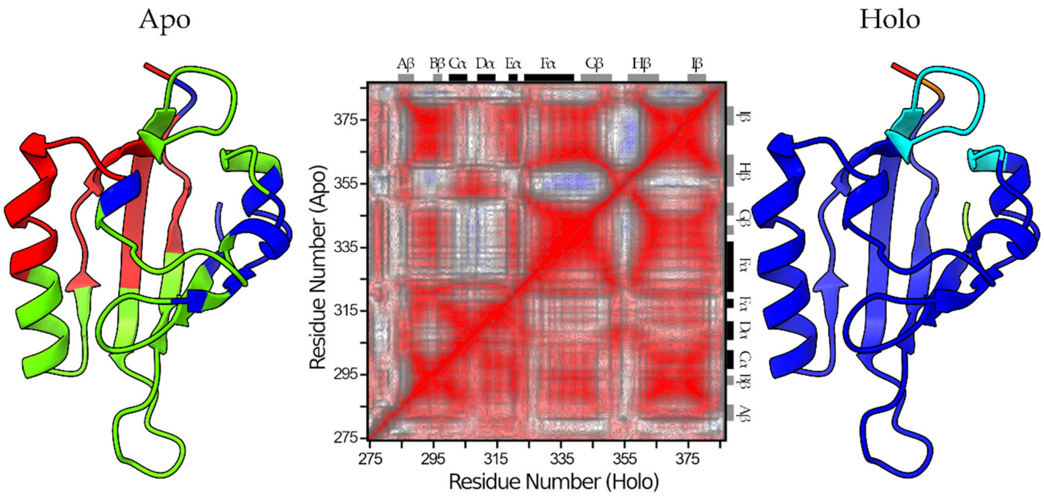

Figure S5. Representation of communities for residues belonging to the AhR PASB domain for apo (left) and holo (right) simulations. Distance cross correlation matrix for the AhR PASB domain (center) is reported with secondary structure annotation shown on top and on the right. Apo simulations are in the in the upper triangle and holo in the lower triangle of the matrix.

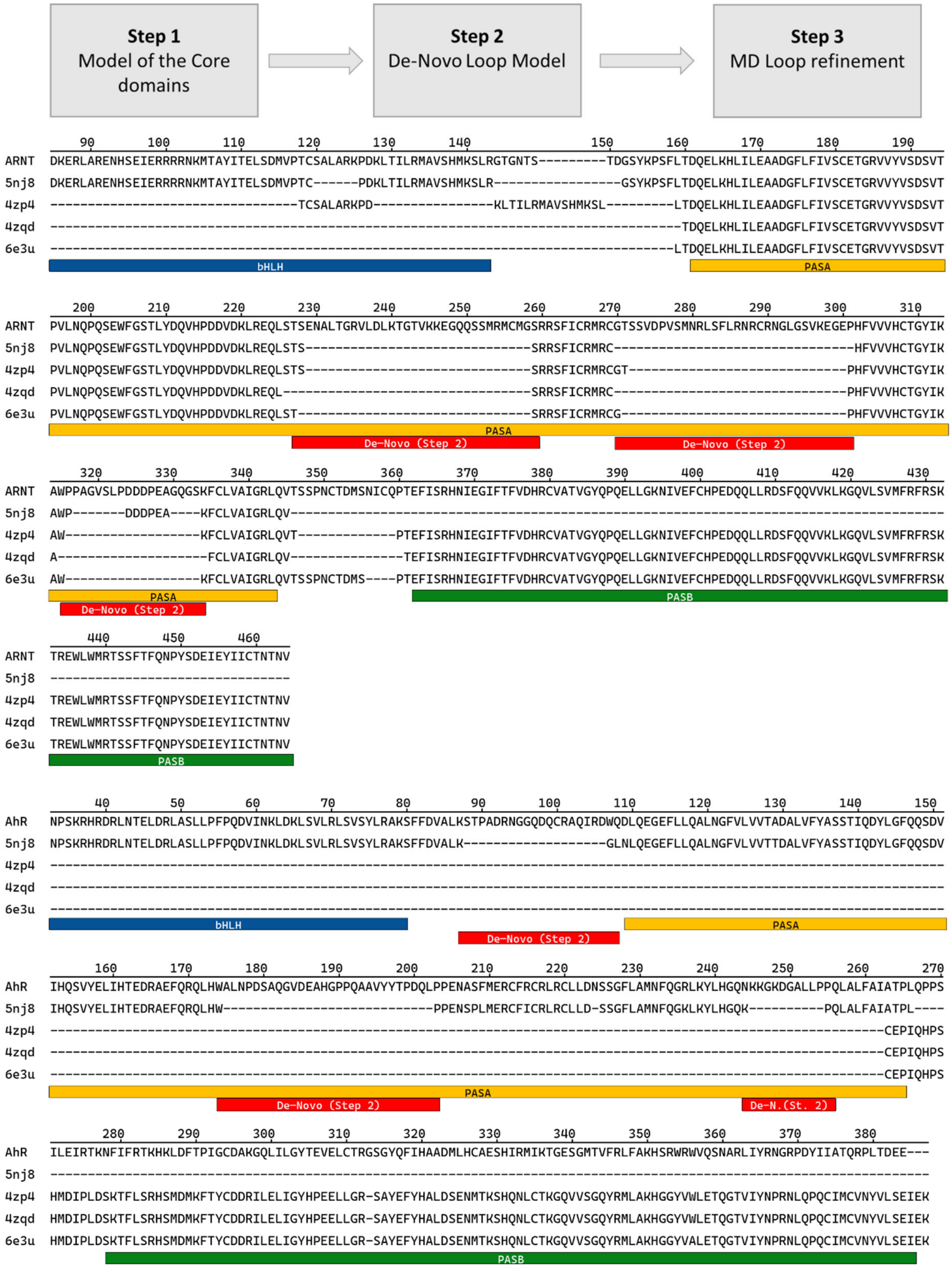

Figure S6. Multiple sequence alignment for the AhR:ARNT complex and the templates used for the homology modelling steps. Structural domains are annotated with blue (bHLH), yellow (PASA) and green (PASB) rectangles. Loops that were modelled de-novo are annotated with red rectangles.

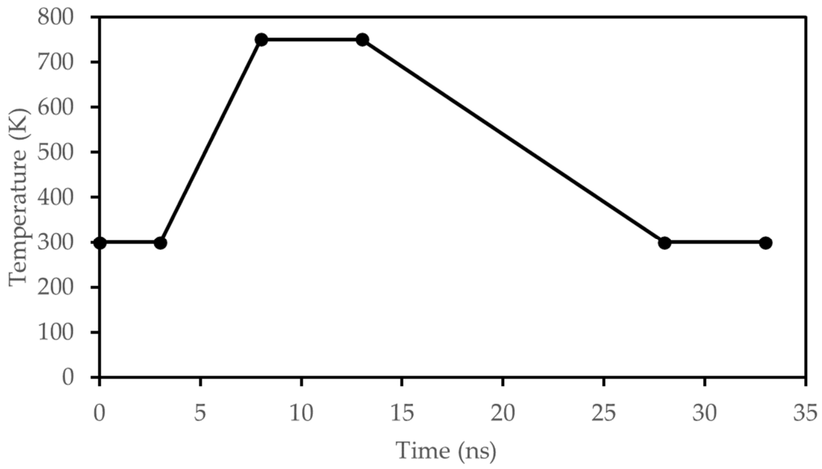

Figure S7. Plot of the simulated annealing protocol. Temperature is increased and then slowly decreased to find a good starting conformation for the protein PASA loops.

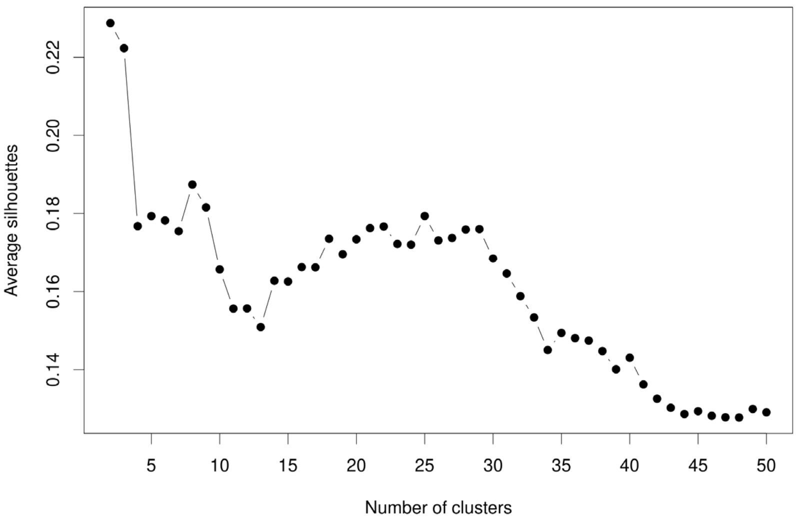

Figure S8. Plot of the average silhouette width against the number of clusters.

Table S1. Atoms involved in distances used to train the SOM.

Atom 1	Atom 2
ARNT ARG88 CZ	DNA DT8 P
ARNT ARG91 CZ	DNA DG9 P
ARNT HIS94 CG	DNA DG9 O6
ARNT GLU98 CD	DNA DC4 N4
ARNT ARG99 CZ	DNA DG7 P
ARNT ARG99 CZ	DNA DC6 P
ARNT ARG101 CZ	DNA DC4 P
ARNT ARG101 CZ	DNA DT3 P
ARNT ARG101 CZ	ARNT GLU98 CD
ARNT ARG102 CZ	DNA DC6 P
ARNT ARG102 CZ	DNA DG5 P
ARNT LYS128 NZ	DNA DT4 P
AhR LYS36 NZ	DNA DG7 P
AhR LYS36 NZ	AhR ASP40 CG
AhR LYS36 NZ	DNA DC6 P
AhR ARG37 CZ	DNA DG2 P
AhR HIS38 CG	DNA DT3 P
AhR ARG39 CZ	DNA DG7 O6
AhR ARG39 CZ	DNA DG7 P
AhR ARG39 CZ	DNA DC6 P
AhR ARG41 CZ	DNA DT3 P
AhR ARG41 CZ	DNA DG2 P
AhR ASN43 CG	DNA DA5 P
AhR LYS65 NZ	DNA DA5 P
AhR LYS65 NZ	DNA DC4 P

.
